# Abnormal Infant Islet Morphology Precedes Insulin Resistance in PCOS-Like Monkeys

**DOI:** 10.1371/journal.pone.0106527

**Published:** 2014-09-10

**Authors:** Lindsey E. Nicol, Timothy D. O’Brien, Daniel A. Dumesic, Tristan Grogan, Alice F. Tarantal, David H. Abbott

**Affiliations:** 1 Pediatric Endocrinology, Wisconsin School of Medicine and Public Health, Madison, Wisconsin, United States of America; 2 Veterinary Population Medicine, University of Minnesota, St. Paul, Minnesota, United States of America; 3 Obstetrics and Gynecology, University of California Los Angeles, Los Angeles, California, United States of America; 4 Department of Medicine Statistics Core, School of Medicine at University of California Los Angeles, Los Angeles, California, United States of America; 5 Departments of Pediatrics and Cell Biology and Human Anatomy, and the California National Primate Research Center, University of California Davis, Davis, California, United States of America; 6 Obstetrics and Gynecology and Wisconsin National Primate Research Center, University of Wisconsin, Madison, Wisconsin, United States of America; Azienda Policlinico S. Orsola-Malpighi, Italy

## Abstract

Polycystic ovary syndrome (PCOS) is prevalent in reproductive-aged women and confounded by metabolic morbidities, including insulin resistance and type 2 diabetes. Although the etiology of PCOS is undefined, contribution of prenatal androgen (PA) exposure has been proposed in a rhesus monkey model as premenopausal PA female adults have PCOS-like phenotypes in addition to insulin resistance and decreased glucose tolerance. PA female infants exhibit relative hyperinsulinemia, suggesting prenatal sequelae of androgen excess on glucose metabolism and an antecedent to future metabolic disease. We assessed consequences of PA exposure on pancreatic islet morphology to identify evidence of programming on islet development. Islet counts and size were quantified and correlated with data from intravenous glucose tolerance tests (ivGTT) obtained from dams and their offspring. Average islet size was decreased in PA female infants along with corresponding increases in islet number, while islet fractional area was preserved. Infants also demonstrated an increase in both the proliferation marker Ki67 within islets and the beta to alpha cell ratio suggestive of enhanced beta cell expansion. PA adult females have reduced proportion of small islets without changes in proliferative or apoptotic markers, or in beta to alpha cell ratios. Together, these data suggest *in utero* androgen excess combined with mild maternal glucose intolerance alter infant and adult islet morphology, implicating deviant islet development. Marked infant, but subtle adult, morphological differences provide evidence of islet post-natal plasticity in adapting to changing physiologic demands: from insulin sensitivity and relative hypersecretion to insulin resistance and diminished insulin response to glucose in the mature PCOS-like phenotype.

## Introduction

Polycystic ovary syndrome (PCOS) is one of the most prevalent endocrine and metabolic disorders in reproductive-aged women, but its etiology is still not understood. PCOS frequently becomes symptomatic in adolescence and has a heterogeneous phenotype, requiring at least two of the following three criteria for diagnosis: hyperandrogenism, oligo-anovulation, and/or ultrasonographic evidence of polycystic ovaries [1]. Other associated physiologic and metabolic abnormalities often include gestational diabetes, impaired glucose tolerance, hyperinsulinemia and type 2 diabetes mellitus, making women with PCOS at significant risk of increased morbidity and mortality [2].

Studies with nonhuman primates (NHP), sheep, rats and mice have suggested an epigenetic mechanism for PCOS based upon *in utero* exposure of the developing female fetus to androgen excess and associated metabolic consequences [3], [4], ; a hypothesis supported by evidence of an altered epigenome in visceral adipose tissue from prenatally androgenized (PA) female NHP [7], [8]. Female rhesus monkeys exposed to prenatal androgen excess have shown the most comprehensive adult PCOS-like phenotype, meeting all three diagnostic criteria in addition to the metabolic comorbidities of insulin resistance, impaired pancreatic beta cell function and increased prevalence of type 2 diabetes mellitus [4]. Interestingly, this adult metabolic phenotype is preceded by excessive insulin sensitivity, increased insulin secretion relative to insulin sensitivity in infancy and increased rapidity of glucose clearance [9].

Such endocrine antecedents of glucose homeostasis in infant PA female rhesus monkeys suggest that experimentally-induced fetal androgen excess may differentially program insulin action and secretion in infancy, preceding the onset of later adult metabolic dysfunction. In support of this concept, pancreatic beta cell development begins in early fetal life in rhesus monkeys [10] during which time regulatory mechanisms governing islet morphology and function may be susceptible to hormonal and metabolic changes *in utero,* particularly since the primate pancreas expresses androgen receptors [11], [12]. In addition, as androgen treatment of monkey dams also induces mild maternal glucose intolerance and transient hyperglycemia in both dams and PA female fetuses [9], quantifying changes in PA monkey islet morphology may elucidate effects of both fetal excess androgen exposure and accompanying fetal glucose dysregulation on islet development, implying an epigenetic origin for abnormal glucose regulation in this well-established, NHP model for PCOS.

The pattern of hyperinsulinism from offspring of diabetic mothers follow by impaired glucose homeostasis is clinically well characterized in humans and has been described before in rodent models [13]. Given the similar pattern in the NHP model for PCOS, we were interested in whether such altered glucoregulatory function had morphological consequences for pancreatic islets in PA monkeys. In this pilot study, we thus quantified pancreatic islet morphology, as a marker for islet development in female monkeys exposed to transient testosterone and mild maternal glucose intolerance while *in utero,* looking for morphological alterations that could be associated with abnormalities of glucose metabolism measured by ivGTT. Given the relative hyperinsulinism of PA monkey infants, we hypothesized that PA infants would demonstrate morphologic changes consistent with islet hyperplasia in response to *in utero* hyperglycemia from maternal androgen treatment, similar to that seen in human offspring of diabetic mothers [14]. We also anticipated finding morphometric antecedents in the PA infant that carried forward into PA adult female pancreatic dysfunction, such as decreased islet number or fractional area providing a link between pancreatic islet development and adult metabolic disease, as recently proposed to precede type 2 diabetes mellitus in humans [15].

## Results

### Experiment 1: Infant morphology

**Figure 1:**
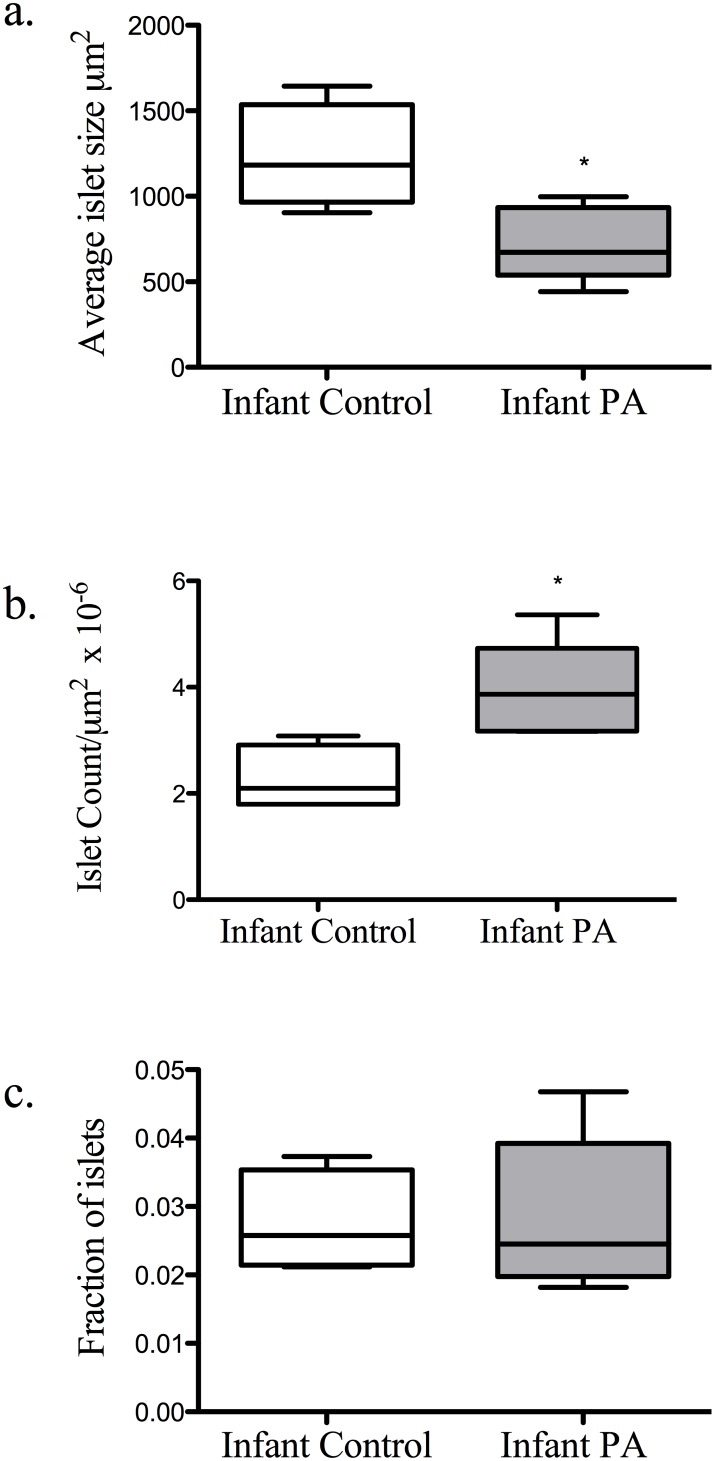
A decrease in average islet area (*P*<0.05) (a) was associated an increase in the islet count (*P*<0.05) (b) preserving the overall islet fractional area (c).

**Figure 2:**
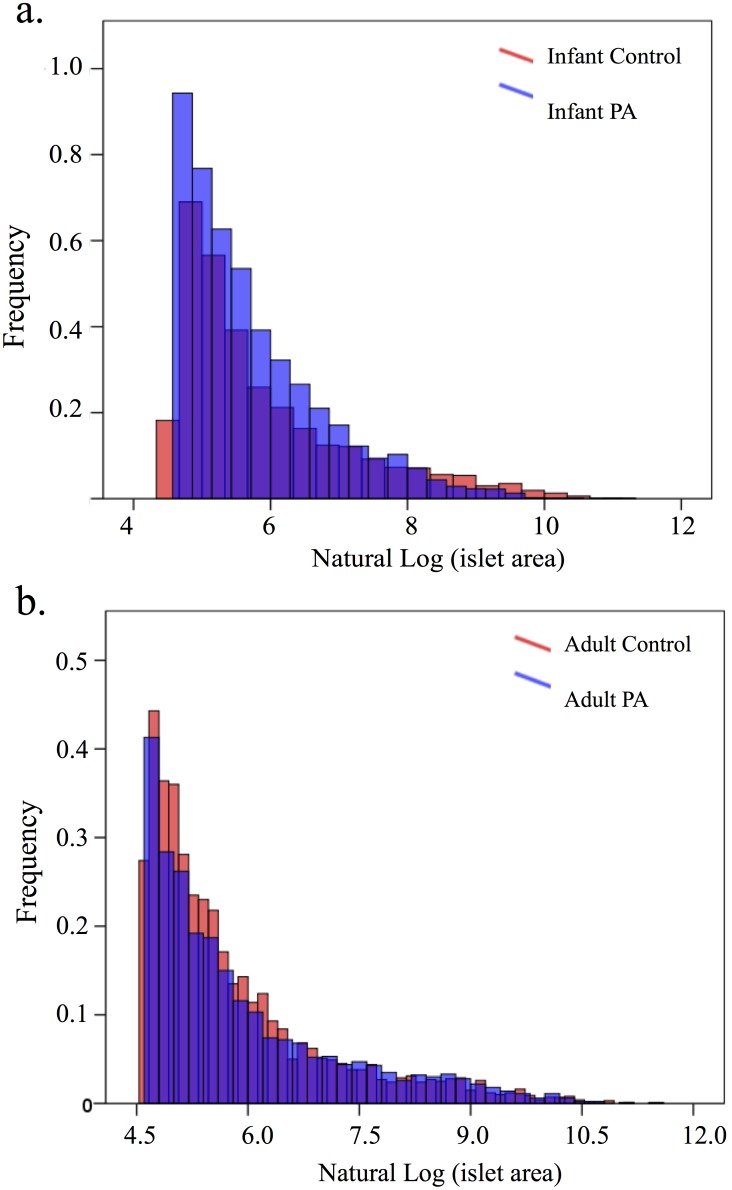
PA female infant islet size distribution is significantly shifted towards an increase frequency of smaller islets (*P*<0.001) (a) and the PA female adults are shifted towards a higher frequency of larger islets (*P*<0.001) (b).

**Figure 3:**
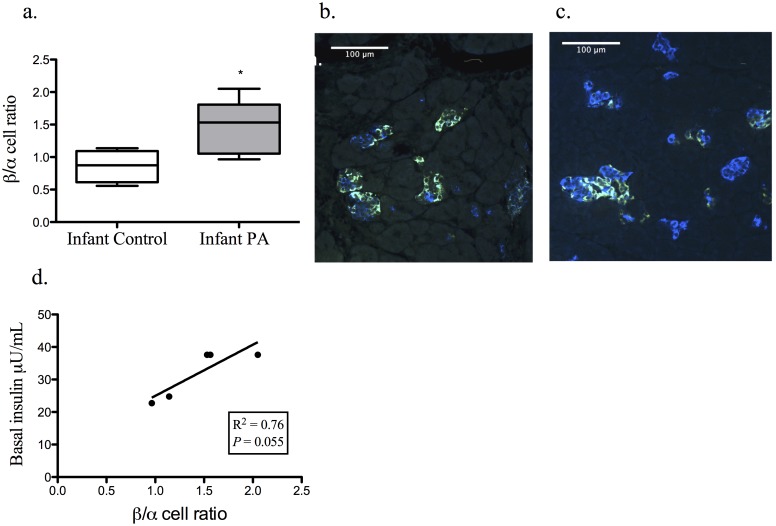
PA female infant islets had an increase in their beta to alpha cell ratio (*P*<0.05). (a) Immunofluorescent images representing the beta (blue) versus alpha (green) cell predominant islets of the PA infant (c) verses controls (b). Regression analysis demonstrates a trend for correlation between increased relative beta cell mass and basal insulin levels (d).

Average islet size by Mann Whitney U-test was decreased *(P<*0.05) in PA infants compared to controls (control 1105 (939, 1498); PA 788 (520, 949) µm^2^), but was accompanied by a corresponding increase *(P<*0.05) in islet numbers (control 2.27 (±0.31); PA 3.94 (±0.40)/µm^2^×10^−6^) preserving fractional islet area (Figure 1a–c). Analysis of the distribution of islet size demonstrated a greater frequency of smaller islets in the PA group (*P*<0.001) (Figure 2a). PA infants also showed an increased proportion of beta to alpha cells (*P*<0.05; control 0.86 (±0.12); PA 1.45 (±0.19)), which tended *(P* = 0.056) to positively correlate with basal insulin levels (Figure 3a–d). The morphologic data (i.e., islet fractional area, islet count/section area, average islet size, and islet size distribution) did not correlate with basal serum glucose or insulin levels, glucose clearance (K_G_), insulin sensitivity, disposition index, (AUC)_glucose_, nor with AUC_insulin_ of the gestational iv glucagon test during the third trimester or the ivGTT at 45 days of life (data not shown).

### Experiment 2: Adult morphology

**Figure 4:**
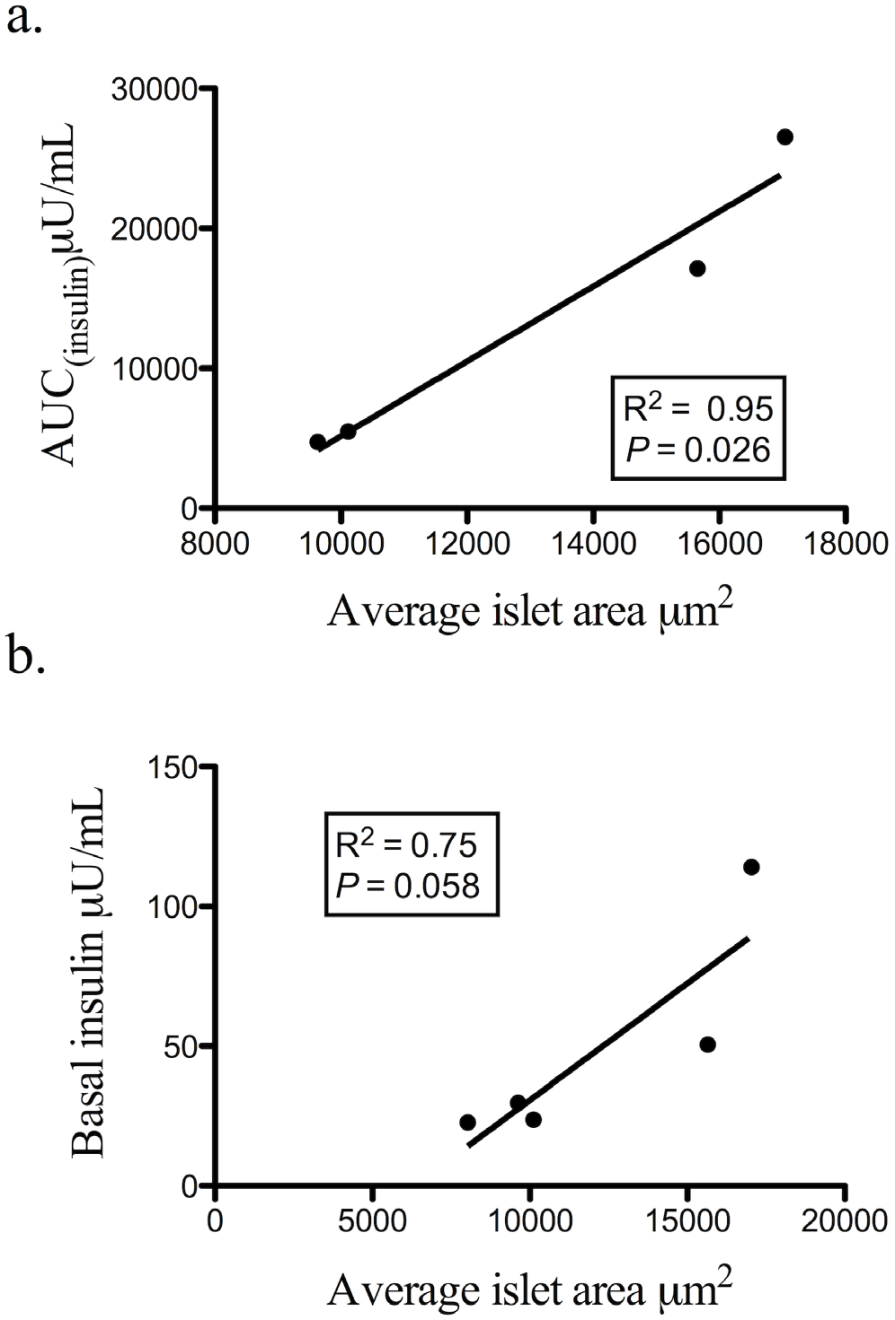
PA adult female average islet size positively correlates with AUC_insulin_ (a) and demonstrates a trend versus basal insulin (b).

In adulthood, PA female pancreatic sections, compared to controls, showed a trend (*P* = 0.067) towards reduced fraction of small islets, ranging from 100 to 1100 µm^2^ in size (data not shown) and decreased islet counts (*P* = 0.068; control 8.25 (±0.75); PA 6.11 (±0.74))/µm^2^×10^−6^) (data not shown). Although the PA females did not otherwise differ in morphologic measurements compared to controls the distribution of islet sizes demonstrated a significant decrease in frequency of smaller islets (*P*<0.001) (Figure 2b). In addition, PA adult pancreatic morphological and ivGTT parameters demonstrated a positive correlation (*P*<0.05) between average islet size versus AUC_insulin_ levels, and a trend (*P* = 0.058) towards a positive correlation with basal insulin (Figure 4a–b), relationships that were absent in adult controls.

### Apoptosis and proliferation markers

Although TUNEL cells were visualized in the exocrine tissue of the pancreas, events within the islets were comparably rare in both the infants and adults and did not differ significantly between PA monkeys and respective controls. Apoptotic events in infant islets were observed in 3 out of 4 controls (median of 0.01 events/islet area) and 1 out of 4 PA infants (0.01 events/islet area). In adults, none of the 7 controls exhibited positive TUNEL staining within the islets, while only 2 out of 6 PA adults showed apoptotic events within islets (median 0.05 events/islet area).

**Figure 5:**
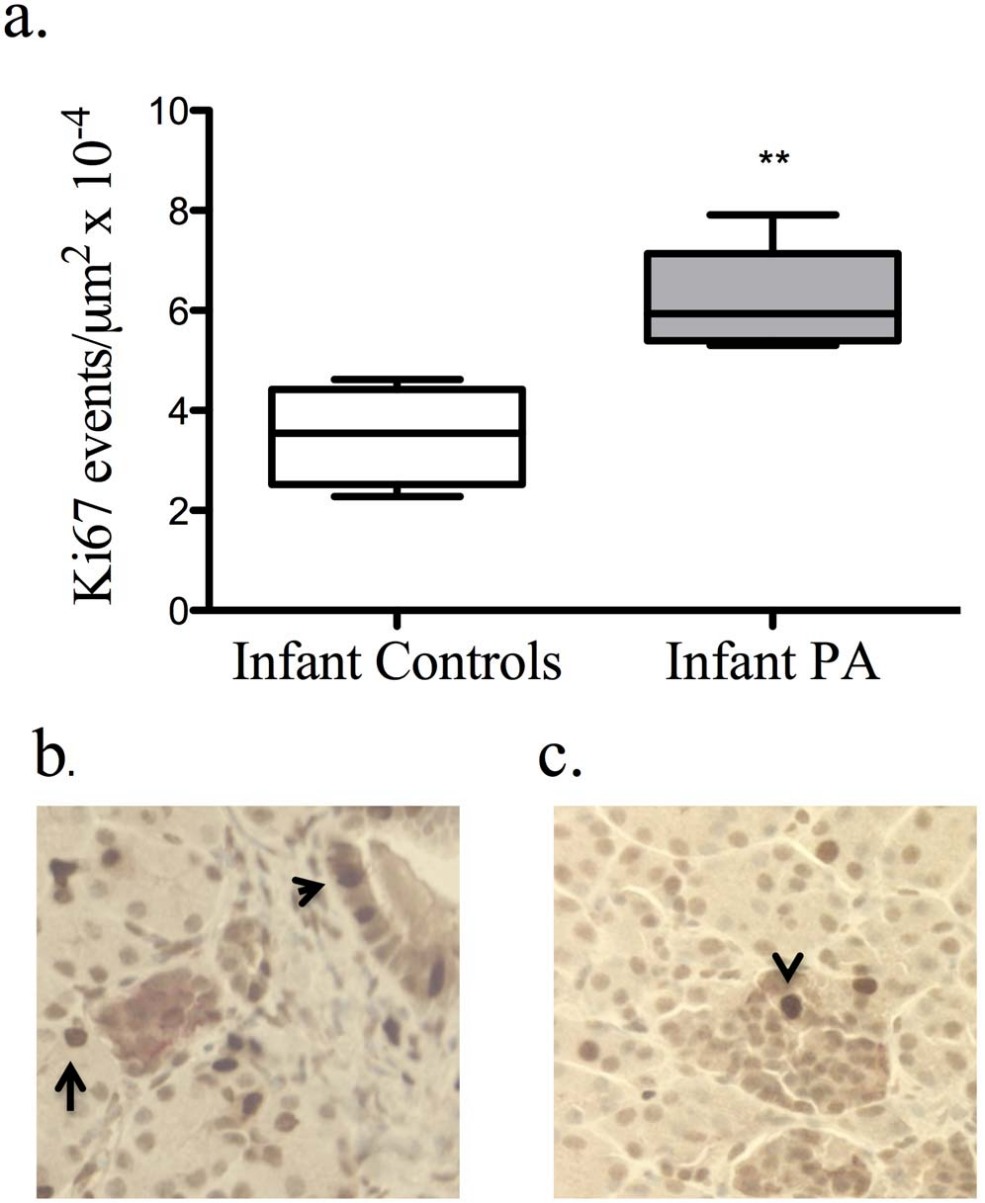
Ki67-positive cells were increased (*P*<0.01) in the islets of PA infants. (a) Proliferative events were seen in both exocrine (arrows) and endocrine tissue (arrow head) of controls (b) and PA (c) infants.

The Ki67 marker for proliferation was seen in both the exocrine tissue and within islets. The number of Ki67 positive cells within islets was greater (*P*<0.01) in PA infants (6.2 (±0.47)×10^−4^/µm^2^) compared to controls (3.5 (±0.49)×10^−4^/µm^2^) (Figure 5), while the number of events in adults was low and did not differ between treatment groups (adult control: 0.14 (±0.043)×10^−4^/µm^2^, adult PA: 0.15 (±0.054)×10^−4^/µm^2^).

## Discussion

While the data presented in this pilot study are constrained by low animal numbers, an inherent problem in the use of NHP models, our results are congruent with a growing body of evidence that links altered fetal environment to adult metabolic disease, specifically abnormal glucose regulation. TP-injected rhesus monkey dams had a transient, postprandial hyperglycemic maternal state, inducing a correspondingly hyperandrogenemic and transient hyperglycemic intrauterine environment for female fetuses [9]. As a metabolic consequence, PA offspring demonstrated over-compensated insulin responses to glucose in infancy [9] and we now demonstrate initial evidence for morphological alterations in PA monkey pancreatic islets.

### Experiment 1: Infants

The islet morphology changes in infant PA monkeys result from a multi-factorial intrauterine exposure: namely, androgen excess and hyperglycemia neither of which can be assessed independently in these experiments, but both can be argued to play a potential role in islet programming. [16] [17] [11], [12], [18].

The stimulatory effects of glucose on beta cell proliferation and growth have been well reported [19], [20]. Elevated glucose levels in PA female fetal monkeys, from diminished maternal pancreatic adaptation to pregnancy due to testosterone-induced insulin resistance [9], may contribute to fetal beta cell proliferation. The morphologic result of such a hyperglycemic gestational environment has been associated with beta cell hyperplasia and islet hypertrophy similar to that seen in offspring of diabetic mothers [14]. PA monkey infants in this study, however, have a smaller average islet size. What apparently compensates for the lack of observed islet expansion, as measured by islet fractional area, is increased islet number that accompanies a greater proportion of beta cells within each islet. Together, these findings are consistent with a compensatory response of developing islets to increases in glucose and testosterone exposure that results in increased proliferation of islets containing a greater proportion of beta cells, but with diminished overall size. Interpreting such PA infant monkey pancreatic changes as a compensatory response to their gestational environment is further supported by the positive correlation between beta to alpha cell ratio and basal insulin levels in PA infants, linking morphologic changes with physiologic phenotype. The lack of difference between PA and control infants with regard to TUNEL positive islet cells suggests a postnatal adaptation of delayed or restricted expansion rather than a destructive apoptotic process. Such plasticity of islet growth in response to the glucose environment has been well described [20], [21], [22]. Proliferation of small islets in PA infant monkeys, however, may reflect a more primate-specific response to glycemic challenge that may be analogous to human maternal small islet proliferation in response to the glycemic challenge of pregnancy [23], a response that is not emulated in rodents but may have some relevance in PA female sheep [18].

In addition to the effects of glucose, there is also sufficient evidence to support the pancreas as sex-steroid responsive [16], [18]. The primate pancreas expresses androgen receptors [11], [12], allowing for a mechanism by which excessive androgen may influence pancreatic development. And *in vitro* studies have demonstrated impaired response to glucose in isolated islets treated with testosterone [17]. Additionally, the PA ovine model also confirms both the expression of androgen receptors in the fetal pancreas as well altered islet development with fetal testosterone exposure [18]. Examining the fetal pancreas from pregnant ewes injected with testosterone, Rae and colleagues demonstrated increased beta cell number and hypersecretion of insulin in the female fetal pancreas. Interestingly, the dams were reported as having normal glucose tolerance suggesting a direct role of androgens separate from the effects of maternal glucose, but this was not confirmed by direct fetal injections of androgen [6].

### Experiment 2: Adults

Compared to controls, PA adult female monkeys demonstrate impaired glucose regulation [24], [25] and our current data associates this pancreatic impairment with a positive correlation between serum insulin levels and islet size, notably between AUC_insulin_ or basal insulin levels and average islet size. Outside of increased insulin production, how beta cells adapt to meet the demands of diminished insulin sensitivity remains debated and is the subject of ongoing research. Components of beta cell neogenesis, hypertrophy, and increased replication have been studied in several animal models and recently assessed during human pregnancy; these studies have shown a state of normal, but relative, insulin resistance [20], [21], [22], [23]. Although adult PA female monkeys exhibit a positive correlation between islet size and basal or AUC_insulin_, these insulin resistant, mildly glucose intolerant adult PA females [25], [26] demonstrate only subtle morphological changes in the pancreas including loss of smaller islets and decreased islet counts that are most pronounced by the frequency of distribution data. Fractional area and ratio of beta to alpha cells are comparable to adult female monkey controls, and there is no evidence of increased apoptosis or proliferation to suggest deficits in beta cell replication or turnover, respectively. This altered size distribution despite maintenance of fractional islet area, however, suggests reduced regenerative islet capacity accompanies glucoregulatory impairment. Subtle PA monkey pancreatic islet adaptation may thus result in PA monkey-specific correlations between adult pancreatic islet morphology and basal and glucose-stimulated circulating insulin levels. Both the shift towards a distribution of larger islets and the correlation of increasing islet size with increasing insulin levels in the PA female adult monkeys suggest that in their hyperandrogenic, insulin resistant state there are fewer but larger islets responsible for the insulin production. This leads to the question of whether such pathophysiology is a potential nidus for beta cell dysfunction and eventual burnout and maybe analogous to a pre-diabetes state in humans [15].

Although answering such inquiries will require further investigation the identified discrete changes in infant and adult islet morphology within these current experiments highlight important impacts on islet development that are both congruent with prior studies as well as novel in its applicability to human disease. Murine studies have demonstrated severely limited beta cell proliferation with advancing age [27], [28]. The lack of increased islet numbers in our hyperinsulinemic PA female adults illustrates this as well; the adaptation to produce more insulin appears to come from a distribution of larger islets rather than an increased number or area. The almost complete absence of apoptosis in these same pancreata, however, attests to the resilience and long life-span of beta cells that has also been previously reported [29]. Additionally, our present study on altered PA monkey islet morphology adds to the previous human findings that maternal metabolic dysregulation imparts measurable outcomes in offspring in regards to pancreatic morphology and function. Our data add to the growing evidence supporting the clinical importance of optimizing maternal metabolic health to prevent the programming of future metabolic disease in the next generation.

## Materials and Methods

### Ethics statement

No live animal work was conducted in the current study. Monkey pancreata harvested at both the California and Wisconsin National Primate Research Centers (CNPRC (Experiments 1) and WNPRC (Experiment 2), respectively) were stored at WNPRC before processing for immunohistochemical analyses at both WNPRC and Oregon Health Sciences University (OHSU). Some of the adult pancreata were obtained through the WNPRC’s Nonhuman Biological Material Distribution program. The WNPRC principal investigator of the studies that originally obtained the monkey pancreata (D.H. Abbott) gave permission for fellow authors to use harvested samples for this study. The care and housing of, and harvesting of tissues from, female rhesus monkeys (*Macaca mulatta)* at both the California and Wisconsin National Primate Research Centers (CNPRC (Experiment 1) and WNPRC (Experiment 2), respectively) were fully compliant with the recommendations of the Guide for the Care and Use of Laboratory Animals and the Animal Welfare Act. All procedures were approved prior to implementation by the Institutional Animal Care and Use Committees at the University of California, Davis and the Graduate School, University of Wisconsin, Madison.

### Samples

Experiment 1 (CNPRC): Pancreata was assessed from a total of nine female infants (∼2 months of age). Five were born from dams receiving 15 mg of testosterone propionate (TP) for 40 days starting on day 40 of gestation [30], and four from control dams of similar age and body weight receiving identically timed oil vehicle injections (total N = 9). In both Experiments 1 and 2, the TP dosing schedule elevated circulating testosterone levels in fetal females to those typically found in fetal males during the early stages of pancreatic organogenesis [4], [30]. Female infants were delivered by Cesarean-section at term (160±2 days gestation) [30], at which time body weight, crown-rump length, and assessments of the placenta were found to be within normal limits [30]. The infants were nursery reared using established methods for housing and diet for this age group [31]. TP-treated dams showed mild-to-moderate glucose intolerance during the second trimester (∼80 days gestation; term 165±10 days), while PA female infants at 45 days postnatal age showed increased insulin sensitivity and disposition index [9]. The harvesting of infant pancreatic tissue was accomplished following pre-medication with ketamine, at least 15 mg/kg injected intramuscularly, and induction of a deep plane of anesthesia with an intravenous injection of barbiturate (25–50 mg/kg) to effect (stoppage of the heart) prior to necropsy.

Experiment 2 (WNPRC): Pancreata from was assessed from thirteen sexually mature, premenopausal female rhesus monkeys. Six PA females were born from dams who received daily subcutaneous (s.c.) injections of 10 mg TP for 15–35 days starting at 42±2 days gestation [4], [32] and seven from untreated controls of similar age and body weight (total N = 13). Adult PA offspring develop a PCOS-like phenotype, insulin resistance, and increased incidence of type 2 diabetes mellitus, as previously reported [24], [33]. Prior to the current investigation, these adult females were involved in a series of mechanistic studies, including [16], [18], [19], [20]. The animals were fed Purina monkey chow (product no. 5038, Ralston Purina, St Louis, MO, USA) twice daily and supplemented with fresh fruit, vegetables and browse materials as part of the environmental enrichment program. The formulation of monkey chow provided 70% of calories as carbohydrate, 13% as fat, and 17% as protein, with a high fiber content. Adult females were pair housed with an adult female conspecific in standard laboratory housing (5.6 to 8.0 sq. ft.), unless incompatible with available partners. The housing environments were maintained on a 12∶12 light cycle, and were equipped with structural enhancements of perches, toys and other manipulanda, providing ad libitum access to water. Tissue harvests were performed as described for the infants in Experiment 1 and tissue sections were evaluated from 6 PA premenopausal (menopause transition in rhesus monkeys, 26–28 years of age [34]) adult females (age: 22.4±2.4 years, *P*<0.11 vs. controls; weight: 7.3±1.3 kg, *P*<0.013 vs. controls, mean±SEM) and 7 adult female untreated controls (age: 19.4±3.4 years, weight: 9.6±1.5 kg).

### Glucoregulatory assessment

Glucoregulatory assessments were performed after overnight fasts as previously reported on dams at 80 days of gestation, infants at 45 postnatal days and on adults during the mid-to-late reproductive years before menopause [9], [24], [25], [35] (Table 1). Basal insulin and glucose, insulin sensitivity (SI), disposition index (DI), and area-under-the-curve for insulin (AUC_insulin_) and glucose (AUC_glucose_) were measured. TP-treated dams showed mild-to-moderate glucose intolerance, while PA infants had increased insulin sensitivity and disposition index [5]. In adulthood, the PA group had decreased insulin sensitivity and disposition index [33]. There were no changes in fetal insulin and glucose responses to glucagon challenge [9]. Although this data has been published previously we have included a summary of the measured insulin parameters referenced in our figures for our cohorts (Table 2).

**Table 1:** Composition of ivGTT by age.

ANIMAL	AGE	ivGTT COMPOSITION	TOLBUTAMIDE (at 20 minutes)
**Dams**	80 days gestation	300 mg/kg glucose	No
**Infant**	45 postnatal days	500 mg/kg glucose	20 mg/kg
**Adult**	Premenopausal	300 mg/kg glucose	20 mg/kg

**Table 2:** Insulin levels (mean±SEM) in infants on postnatal day of life 45 and premenopausal female adults.*

Age group	Basal insulin(µU/mL)	AUC_Insulin_(µU/mL*min)
Infant control	4.35 (±0.97)	1216 (±129)
Infant PA	8.98 (±4.11)	897 (±122)
Adult control	31.69 (±7.11)	16794 (±5752)
Adult PA	48.14 (±17.22)	13479 (±5202)

*The ivGTT parameters have been previously published for female adults demonstrating decreased insulin sensitivity and disposition index [24], [33] and for infants reporting increased insulin sensitivity and disposition index [9]. The insulin levels in this table have been derived from the referenced data to reflect the cohort of insulin values used to correlate with morphologic parameters (Figures 3 and 4). There are no significant differences between age-contemporary treatment groups for the cohort analyzed.

### Histology

#### Immunofluorescence study

Infant and adult pancreatic tissues (from the tail region) obtained at harvest were stored at ≤−80°C until processed for immunostaining. Two randomly selected samples from each animal were thawed directly in 4% paraformaldehyde at 4°C and processed for paraffin embedding and histologic sectioning by conventional methods. Each sample was sectioned at 7 µm.

Two samples from each infant and adult monkey were incubated overnight with rabbit anti-insulin polyclonal antibodies at a 1∶1000 dilution (#20056, Immunostar, Hudson, WI) and guinea pig anti-glucagon polyclonal antibodies at a 1∶7000 dilution (#4031-01F, Linco, St. Charles, MO), after deparaffinization, rehydration in serial ethanol washes, heat retrieval in citrate, and blocking with 5% normal goat serum. Islets were visualized using goat anti-rabbit 633 and goat anti-rabbit 488 at 1∶500 dilutions demarking the alpha and beta cells of the islets. Negative controls were developed with the same protocol less the addition of the primary antibodies. Six by six fields were imaged at 10X magnification and tiled together to create one digital image using the Nikon A1 confocal microscope which incorporated the entire cross section of the sample. Image J was used to quantify total section area (excluding attached connective tissue and large vascular structures), islet count, and each islet area. Alpha and beta mass was also quantified using Image J on the corresponding single channel images.

The data obtained from both sections of each animal was summed for semi-quantitative analysis. An average of 541 islets were quantified for each adult and 813 from each infant. The fractional area was calculated by dividing the total islet area (of all islets combined for the relevant pancreatic section) by the total area quantified for that section; the islet count was defined as total number of islets per section area. Average islet size equaled the total islet count divided by the total islet area. Cut off value for smallest islets counted was set at 100 µm^2^ or greater to eliminate two and three beta-cell aggregates.

ApopTag Fluorescein *In Situ* Apoptosis Detection Kit (#S7110, Millipore, Temecula, CA) was used for quantification of apoptosis in the islets using the manufactures protocol for fluorescent staining of paraffin-embedded tissue. Sections were double labeled with insulin using rabbit anti-insulin antibodies at 1∶1000 (#20056, Immunostar, Hudson, WI) and goat anti-rabbit secondary 633 (#A21071, Invitrogen) at 1∶500 dilution to help identify events that occurred within in the endocrine verses exocrine tissue. Negative controls were developed with the same protocol less the addition of the primary antibodies. Two sections from each animal were imaged using laser-scanning confocal microscope (Zeiss LSM710) at 20X. Twenty-five fields at this magnification, arbitrarily starting at the gross center of the section, were tiled together into one image using the Zeiss processing software. The number of apoptotic events were manually counted and divided by total surface area scanned using Image J. Mouse monoclonal anti-human Ki67 (#M7240, Dako, Carpinteria, CA) 1∶75 dilution was used to quantify proliferation events. Sections were deparaffinized in xylene, rehydrated in serial washes of ethanol, heat treated in citrate buffer and blocked using 5% bovine serum antigen and 5% normal goat serum for one hour. The anti-Ki67 antibody was incubated overnight at 4°C. Then visualized using Vectastain Elite ABC peroxidase based detection system using DAB (#PK-4002, Vector, Berlingame, CA). Sections were subsequently incubated with the same insulin antibodies noted above at 1∶1000 overnight at 4°C and developed using Vectastain ABC alkaline phosphatase detection kit (AK-5001) and Vector Red (SK-5100) to help localized events to the endocrine tissue. Negative controls were developed with the same protocol less the addition of the primary antibodies. Digital images were taken using Leica Application Suite V3.6 software under bright-field microscopy at 20X on the Leica DFC340FX. Five random, non-overlapping images containing at least one islet were quantified from two different tissue samples for each animal. Like the TUNEL assay, events were manually counted in Image J and divided by quantified islet area.

### Statistical evaluation

Islet fractional area and islet count were assessed using a two-side t-test and a *P* value<0.05 as significant. Data were reported as means (± standard error of the mean (SEM)). Given that islet size is not normally distributed, average islet size was analyzed by non-parametric Mann-Whitney U testing and reported as median (25%, 75% quartiles). Regressions illustrate the best fit line from a data series representing the mean morphologic parameter of each animal versus corresponding values for ivGTT (infant PA and controls: postnatal day 45 [9]; dams of infant subjects: second trimester, 80 days of gestation; adult PA females: 20.3 (±1.4) years; adult controls: 22.3 (±1.0) years (mean (±SEM)) or for an iv glucagon test (fetus: third trimester, 140 days of gestation [9]. The empirical cumulative distribution function (ECDF) plotted for each treatment and control group was constructed by the probability of islets size being less than or equal to the given value. The distributions of the islets were transformed using the natural log transformation for visualization purposes (Figure 2). Statistical analysis of the distribution data was performed using 2-sample Kolmogorv-Smirnov test.
